# Medical Observations and Inquiries. Vol. VI

**Published:** 1785

**Authors:** 


VI. *Medical Obfervations and Inquiries.* Vol. VI.

(Continued from page 218.)

IX. *REMARKS on the Cure of the Epilepfy; to which are added fome Confiderations on the Practice of Bleeding in Apoplexies. By* John Fothergill, M. D. F. R. S. — Thefe remarks on the epilepfy, written towards the clofe of a long and extenfive practice, tend rather to increafe our doubts concerning the efficacy of the remedies hitherto employed in this difeafe, than to point out any other and more fuccefsful means of relief. Dr. Fothergill, confidering a diftended ftomach and loaded bowels as the moft general difpofing caufes to this difeafe, lays his chief ftrefs in the treatment of it on a fpare diet, and the prevention of coftivenefs. He even goes ſo far as to query whether valerian, caſtor, fœtid gums, and other diſguſtful medicines, commonly preſcribed in epileptic caſes, do not occaſionally produce a good effect merely by leſſening the patient's appetite.—The remarks on the practice of bleeding in apoplexies, with which the paper cloſes, are intended to inculcate great caution in the uſe of this remedy. Dr. Fothergill is of opinion that the pulſe in theſe caſes is often an inſufficient guide: it may be, he obſerves, that ſtruggle which ariſes from an exertion of the *vis vitæ* to reſtore life, and it is poſſible that, by a copious bleeding, the animal ſtrength may be ſo much reduced, and the effort begun ſo powerfully checked by the evacuation and the effects of the diſeaſe itſelf, that the patient may expire ſoon afterwards, or ſurvive a few days, and ſuffer a hemiplegia; none of which might probably happen were bleeding to be omitted. As he ſuppoſes the diſeaſe to be moſt commonly occaſioned by a large undigeſted meal, diſtending the ſtomach, preſſing upon the aorta, obſtructing the free expanſion of the lungs, and of courſe increaſing the determination of blood to the head, he is of opinion that inſtead of reducing ducing the patient's ſtrength by blood-letting, we ſhould evacuate the ſtomach and inteſtines by emetics, purgatives, and irritating clyſters, and at the ſame time excite ſo plentiful a flow of blood and vital energy to the lower extremities as we can, by means of ſinapiſms and other ſtimulant applications. Of the propriety of this doctrine, however, though delivered by ſo experienced and reſpectable a phyſician, we cannot refrain from expreſſing our doubts. Exceſs or irregularity in diet may certainly very often be conſidered as an occaſional cauſe of the diſeaſe in theſe caſes, and of courſe the neceſſity of ſpeedily emptying the bowels muſt be obvious; but whether emetics will not tend, by their operation, to increaſe the determination to the head, and of courſe to do harm, is a point, perhaps, not ſo clearly decided. We muſt own, therefore, that we ſhould be tempted in a caſe of this ſort to give the preference to purgatives and clyſters. But in the mean time we ſhould be unwilling to neglect the chance of relief which the opening a blood veſſel, by unloading the veſſels of the brain, ſeems to offer in caſes of this kind, where the more immediate cauſe of the diſeaſe may, in the greater number of inſtances, perhaps, be,

 with with good reaſon, aſcribed to a congeſtion of blood within the head. *

Dr. Fothergill very judiciouſly cautions perſons, who, from their make, may ſeem more diſpoſed to apoplexies than others, againſt looking backwards any length of time, without turning the whole body. He obſerves that if we take a hollow flexible tube of leather, ſix inches or more in length, holding one end in each hand, and endeavour, by turning each hand a contrary way, to twiſt the tube, we ſhall make but very little impreſſion on its cavity, whilſt our hands are at that diſtance, with one twiſt of our hands; but that if we ſhorten the diſtance, and leave only one or two inches between each hand, the ſame turn of the hands, oppoſite ways, will leſſen the diameter of the tube extremely, ſo as almoſt wholly to prevent the paſſage of any fluid. "In ſome reſpects," remarks our author, "the ſame thing happens "to the jugular veins in very ſhort-necked "people, the carotid arteries lying nearer the "center of motion, are very little affected by "the turn of the head even in very ſhort-"necked people, but continue to convey full

* The practice of bleeding in apoplexy is very judiciouſly defended by Dr. Chandler in a work lately publiſhed.— See page 221 of the preſent volume.

"ſtreams

" ſtreams of blood to the head. But this is " not the caſe with the jugular veins; they " lie near the ſurface, and if the neck is ſhort, " and full at the ſame time, the twiſt ſo far " contracts their diameters, that it is impoſſi- " ble for them to return a proportionable quan- " tity. Hence, therefore, firſt a giddineſs; " at length a total, though temporary, ceſſa- " tion of every faculty, or, in other words, a " perfect apoplexy." All this is illuſtrated by the caſe of a gentleman with a very ſhort neck, who, in croſſing the Thames in an open boat, kept his eye ſtedfaſtly fixed on a ſhip, that had formerly belonged to him, for ſome time after he had paſſed it, till at length he loſt himſelf, and ſunk down in the boat.

X. *An encyſted watery Tumor, adhering to the poſterior part of the Bladder, and to the whole length of the Rectum, which brought on a fatal Suppreſſion of Urine. By* Thomas Gery Cullum, *Surgeon at St. Edmond's Bury, in Suffolk, and Member of the Corporation of Surgeons in London.*—The ſubject of this caſe, which is extremely curious, was a young man about eighteen years of age, who had laboured under a ſuppreſſion of urine three days when our author firſt ſaw him. The abdomen was very tenſe and painful to the touch, and the bladder ſeemingly diſtended two inches above the navel. Mr. Cullum, on paſſing his finger up the *anus*, (which was much dilated) felt, as he imagined, the bladder exceſſively diſtended. Finding it impoſſible to introduce a catheter beyond the proſtate gland, he punctured the bladder above the os pubis. Three pints of urine were immediately diſcharged, and in a few hours at leaſt two quarts more had drained away through the canula. The patient was much relieved, yet the tumor of the abdomen ſtill reached above the navel, and that which was felt in the rectum was not ſenſibly diminiſhed. Two days after this operation, the canula having ſlipped out, a ſecond puncture became neceſſary. In about ten days after this ſecond operation, he began to complain of pain all over his body, particularly in his legs, attended with rigors and ſickneſs, and about a fortnight before he died, turning himſelf rather in a hurry in the night, he complained of great pain, and in the morning half a pint of pus came away through the canula, and in three or four hours half a pint more. From that time till his death, which happened thirty-one days after the firſt operation, matter, either by itſelf or mixed with urine, was continually diſcharged. On diſſection, ſeveral encyſted tumors, each as large as an hen's egg, were obſerved adhering to the omentum, and on removing this viſcus, a large cyſt (nearly as big as a quart bottle without its neck) preſented itſelf very much diſtended with a watery fluid, not connected with any thing at top, or at its anterior part, for ſome inches; but backwards, ſtrongly adhering to all the parts contained in the pelvis, and nearly filling the whole cavity of it. Two or three inches below the navel the bladder came to view, on the fore part of this preternatural body, quite collapſed, and containing only two or three large ſpoonfuls of urine. The kidneys were of twice their natural ſize, and in the pelvis of one of them was a ſpoonful of pus. From this ſource our author ſuppoſes the matter diſcharged through the canula to have been derived.

Mr. Cullum very candidly expreſſes his regret that he did not puncture the bladder through the rectum rather than above the os pubis, as the diſſection proved that in this caſe no other operation than the puncture through the rectum could have afforded any chance of relief.

He found, on inquiry, that this diſeaſe had been of ſix years ſtanding, and he has endeavoured voured to ascertain the marks by which a similar complaint may be distinguished: these are a tumor, first, within the sphincter ani; a great distension of the abdomen even before any suppression of urine has come on; the undiminished bulk of the tumor in the rectum and abdomen, even after the bladder has been emptied; and lastly, the circumstance of the patient's being able to bear a strong pressure on the abdomen with little or no pain.

XI. *Remarks on that Complaint commonly known under the Name of the Sick Head-Ach. By* John Fothergill, *M. D.*—This complaint, although it has not been so entirely overlooked, as Dr. Fothergill supposes, by systematic writers, Sauvages having mentioned it under the name of *Vomitus Cephalalgicus*, yet has certainly never been so fully considered, or so clearly descri-bed, as in the paper before us. It is not the complaint, we are told, of any particular age, or sex, or constitution, or season, but is incident to all. Sedentary persons, however, and they who are incautious respecting diet, and of a costive habit, are said to be the most exposed to it. The patient usually awakes early in the morning with a head-ach, which seldom affects the whole head, but one particular part of it, most moſt commonly the forehead. With this is joined more or leſs of ſickneſs. The duration and violence of this complaint is very different in different perſons; in ſome, it goes off in two or three, in others it will laſt twenty-four or more hours. In young perſons it moſt commonly goes off ſoon; but if it continues to harraſs them many years, as it ſometimes has done, the fit, our author obſerves, continues longer, and leaves the whole frame in ſo weak a condition, as to require ſome length of time for the patient to recover. Its returns are very irregular; ſome have it every two or three days, others only once in as many or more months.

Dr. Fothergill's opinion of this complaint is, that, for the moſt part, it proceeds from an inattention to diet; and in the treatment of it his chief reliance is on regimen. This leads him to offer ſome general reflections on the dietetic part of phyſic.

[*To be continued.*]

