# Errata

**Published:** 1785

**Authors:** 


					p. 333,. 1. 22} for and the health, readJb far as relates it
the health.?

				

